# Inverse Association of Vitamin C with Cataract in Older People in India

**DOI:** 10.1016/j.ophtha.2011.03.016

**Published:** 2011-10

**Authors:** Ravilla D. Ravindran, Praveen Vashist, Sanjeev K. Gupta, Ian S. Young, Giovanni Maraini, Monica Camparini, R. Jayanthi, Neena John, Kathryn E. Fitzpatrick, Usha Chakravarthy, Thulasiraj D. Ravilla, Astrid E. Fletcher

**Affiliations:** 1 Aravind Eye Hospital Pondicherry, Aravind Eye Care, Pondicherry, India; 2 Dr. Rajendra Prasad Center for Ophthalmic Sciences, All India Institute of Medical Sciences, New Delhi, India; 3 Centre for Public Health, School of Medicine, Dentistry and Biomedical Sciences, Queen's University Belfast; 4 Dipartimento di Scienze Otorino-Odonto-Oftalmologiche e Cervico Facciali, Sezione di Oftalmologia, Universita‘ degli Studi di Parma, Parma, Italy; 5 Faculty of Epidemiology and Population Health, London School of Hygiene and Tropical Medicine, United Kingdom; 6 Centre for Vision and Vascular Science, School of Medicine, Dentistry and Biomedical Sciences, Queen's University Belfast, United Kingdom; 7 Lions Aravind Institute of Community Ophthalmology, Madurai, India

## Abstract

**Objective:**

To examine the association between vitamin C and cataract in the Indian setting.

**Design:**

Population-based cross-sectional analytic study.

**Participants:**

A total of 5638 people aged ≥60 years.

**Methods:**

Enumeration of randomly sampled villages in 2 areas of north and south India to identify people aged ≥60 years. Participants were interviewed for socioeconomic and lifestyle factors (tobacco, alcohol, household cooking fuel, work, and diet); attended a clinical examination, including lens photography; and provided a blood sample for antioxidant analysis. Plasma vitamin C was measured using an enzyme-based assay in plasma stabilized with metaphosphoric acid, and other antioxidants were measured by reverse-phase high-pressure liquid chromatography.

**Main Outcome Measures:**

Cataract and type of cataract were graded from digital lens images using the Lens Opacity Classification System III (LOCS III), and cataract was classified from the grade in the worse eye of ≥4 for nuclear cataract, ≥3 for cortical cataract, and ≥2 for posterior subcapsular cataract (PSC). Any cataract was defined as any unoperated or operated cataract.

**Results:**

Of 7518 enumerated people, 5638 (75%) provided data on vitamin C, antioxidants, and potential confounders. Vitamin C was inversely associated with cataract (adjusted odds ratio [OR] for highest to lowest quartile = 0.61; 95% confidence interval (CI), 0.51–0.74; *P=*1.1×10^−6^). Inclusion of other antioxidants in the model (lutein, zeaxanthin, retinol, β-carotene, and α-tocopherol) made only a small attenuation to the result (OR 0.68; 95% CI, 0.57–0.82; *P* < 0.0001). Similar results were seen with vitamin C by type of cataract: nuclear cataract (adjusted OR 0.66; CI, 0.54–0.80; *P* < 0.0001), cortical cataract (adjusted OR 0.70; CI, 0.54–0.90; *P* < 0.002), and PSC (adjusted OR 0.58; CI, 0.45–0.74; *P* < 0.00003). Lutein, zeaxanthin, and retinol were significantly inversely associated with cataract, but the associations were weaker and not consistently observed by type of cataract. Inverse associations were also observed for dietary vitamin C and cataract.

**Conclusions:**

We found a strong association with vitamin C and cataract in a vitamin C–depleted population.

**Financial Disclosure(s):**

The author(s) have no proprietary or commercial interest in any materials discussed in this article.

India accounts for approximately 20% of the global burden of blindness, with cataracts being the principal cause.^1^ Population-based studies have reported high prevalence rates of cataract in India^2–4^ compared with western populations, even when the level of cataract surgery is taken into account. Environmental, nutritional, and genetic factors may be important explanatory factors of these high rates, but to date, there is limited information on these in the Indian setting, particularly on antioxidants (especially vitamin C) considered to play a key role in protecting the lens from oxidative stress.^5^ In a small feasibility study for the current study, we reported inverse associations between cataract and plasma levels of vitamin C and other antioxidants.^6^ The levels of vitamin C in the feasibility study were lower than observed in high-income countries. The India Age-Related Eye Disease Study is a large population-based study conducted in 2 centers in north and south India. Our overall objectives were to examine the prevalence and risk factors for cataract and specifically to investigate vitamin C and cataract.

## Materials and Methods

The study sampling has been described in detail.^4^ Briefly, 7518 people aged ≥60 years were identified from household enumeration of randomly sampled clusters in north and south India in the catchment area of the 2 participating hospitals, Aravind Eye Hospital Pondicherry and Dr. Rajendra Prasad Center for Ophthalmic Sciences, New Delhi, but excluding the cities of Delhi, Gurgaon, and Pondicherry. All participants gave full informed written consent. Illiterate subjects had the information leaflet read to them and provided a thumb impression. The study complied with the guidelines in the Declaration of Helsinki, and ethics approval was received from the Indian Council for Medical Research (ICMR), Research Ethics Committees of the All India Institute of Medical Sciences, Aravind Eye Hospital, London School of Hygiene, and Tropical Medicine, and Queen's University Belfast.

Enumerators collected household and individual sociodemographic and economic data (quality of house, land ownership, crowding, education, occupation, caste, religion). Trained fieldworkers interviewed participants at home with a structured questionnaire that included current and past tobacco use (smoking beedis or cigarettes, chewing, or inhaling), current and past alcohol use (frequency and type of drink), type of cooking fuels and stoves, and current and past outdoor work at different times of the day. Diet was assessed by 24-hour recall. Within 1 week of the home interview, participants were brought to the base hospital for the clinical examination that included anthropometry, blood pressure, an eye examination, and blood sample collection. Anthropometry included measurement of height, weight, and mid-upper arm circumference (MUAC). Blood pressure was measured using the automated Omron HEM 705CP device (Omron Healthcare, Inc., Bannockburn, IL), with 2 measurements each of systolic and diastolic pressure. People were asked to bring medications or nutritional supplements to the hospital, and details were recorded.

## Blood Sample

For each participant, a sample of 15 ml of blood was collected in a shaded room in 2 different Vacutainer tubes (10 mL clotted and 5 ml ethylenediaminetetraacetic acid unclotted). The 10-mL blood sample was kept at room temperature for 1 hour to allow for adequate clotting. The unclotted blood sample was kept in the refrigerator. Within 2 hours of collection, both samples were centrifuged at 3000 rpm at 4°C (using a cold centrifuge) for 15 minutes, aliquoted, and transferred to a −70°C freezer. Samples were subsequently shipped in dry ice to Queen's University Belfast for analysis by reverse-phase high-pressure liquid chromatography for serum lutein, zeaxanthin, β-cryptoxanthin, α-carotene, β-carotene, α-tocopherol, γ-tocopherol, lycopene, and retinol. Total plasma vitamin C was measured using an enzyme-based assay in plasma stabilized with metaphosphoric acid. Assays were standardized against the US National Institute of Standards and Technology standard reference materials and participated in external quality assurance schemes where available. Cholesterol was measured using an enzymatic assay (Randox, Crumlin, UK) on a Cobas-FARA centrifugal analyzer (Roche Diagnostics, West Sussex, UK). A nonfasting sample of capillary blood was assessed for glucose using a reagent strip test and reflectance meter.

## Assessment of Cataract

After pupillary dilation to at least 6 mm, digital slit-beam images of the lens were taken according to a standardized protocol using the Topcon SL-D7 Digital photo slit lamp for nuclear opacities (Topcon, Tokyo, Japan). Retroillumination images of the lens (one focused on the anterior and one focused on the posterior capsule) were taken with the Neitz CT-S digital camera (Kowa Optimed Inc., Torrance, CA) to capture cortical and posterior subcapsular opacities. Lens opacities were graded according to the Lens Opacities Classification System III^7^ in 0.1-unit steps for each opacity up to a maximum of 6.9 for nuclear opacities and 5.9 for cortical and PSC. The training and quality assurance of the photographers and graders have been described.^4^

We defined the type of cataract on the basis of the Lens Opacities Classification System III grade in the worse eye of ≥4 for nuclear cataract, ≥3 for cortical cataract, and ≥2 for posterior subcapsular cataract (PSC). People with any type of cataract based on these criteria or those whose images could not be graded for type of cataract because of dense opacities were included in the definition of any unoperated cataract. People with any unoperated cataract plus those who were pseudophakic or aphakic in either eye were included in the definition of any cataract (i.e., operated plus unoperated cataract).

## Data Preparation and Statistical Analysis

The distribution of plasma vitamin C was left skewed; 30% of samples were classified above zero but below the lowest level of detection of the laboratory (2 μmol/L). Plasma vitamin C was therefore categorized by quartiles (the lowest quartile comprising 30% of the values as described previously). Nutrient intakes of energy and vitamin C were calculated from the individual food items in the 24-hour recall using the ICMR food composition tables.^8^ Dietary vitamin C was adjusted for total energy intake using the residual model of Willett et al^9^ and categorized by quintiles.

Principal component analysis was used to derive a socioeconomic status index (based on caste, landholding, type of roof, and number of rooms in house, excluding kitchen, toilets, and bathrooms). Midday outdoor exposure was summed over job and life periods and categorized by quartiles. Alcohol and tobacco use were categorized as current or never and past. Body mass index (BMI) was calculated as weight (kilograms)/height^2^ (centimeters). We used World Health Organization guidelines for categories of BMI in Asians.^10^ Diabetes was defined as capillary blood glucose ≥200 mg/dL.^11^ Nutritional status was defined from MUAC values as normal (>23 in men and >22 in women), mild malnutrition (22.1–23 in men and 20.1–22 in women), and moderate to severe malnutrition (<22 in men and <20 in women).^12^ Blood pressure was taken as the average of the 2 readings of systolic or diastolic blood pressure.

Statistical analysis was carried out using Stata 10 (StataCorp 2007 Stata Statistical Software: Release 10; StataCorp LP, College Station, TX). We undertook univariable analysis to examine the association of factors expected to be associated with either plasma or dietary vitamin C: age, sex, socioeconomic status, tobacco and alcohol use, diabetes, anthropometry (BMI, MUAC), and household cooking fuels. We used multivariable logistic regression to investigate the association of vitamin C with cataract or type of cataract. In all analyses, the comparator group were those with no cataract or operated cataract (i.e., <4 for nuclear cataract, <3 for cortical cataract, and <2 for PSC, no dense opacities and no aphakia/pseudophakia). We investigated possible interactions between vitamin C and study center because we have previously reported differences, albeit small, in the prevalence of different types of cataract between north and south India.^4^ We used 2 multivariable logistic regression models, one that included an interaction term of study center and plasma vitamin C and one model of both centers together with no interaction term. All analyses took account of the sampling design in the estimation of robust standard errors. We ran preliminary multilevel logistic models taking account of both the cluster sampling and the household structure (57% of participants lived in households in which they were the only participant, 35% lived in households that included 2 participants, and 8% lived in households that included ≥3 participants). These models showed almost identical results to those using logistic regression with robust standard errors. Because of the computational time for multilevel models, we preferred logistic regression.

We examined other blood antioxidants identified on the basis of the literature.^13^ These were lutein, zeaxanthin, α-tocopherol, β-carotene, and retinol. Because α-tocopherol is lipid soluble, we calculated its ratio to cholesterol. Colinearity between antioxidants entered simultaneously in models was checked by variance inflation factors and showed colinearity (variance inflation factor ∼2) between lutein and zeaxanthin. These antioxidants were therefore included separately in models.

## Results

Of 7518 enumerated people, 5871 had a full eye examination, of whom 5702 gave a blood sample. Plasma vitamin C was available in 5638 people (2668 samples from the Dr. Rajendra Prasad Center for Ophthalmic Sciences and 2970 from the Aravind Eye Hospital Pondicherry). Of the participants who attended the eye examination, there were no significant differences by age (*P=*0.1), sex (*P=*0.4), or cataract status (*P=*0.6) for those with vitamin C measurements compared with those without. Any cataract (operated or unoperated) was graded in 4098 participants (72.7%); 811 people had bilateral aphakia/pseudophakia. Most unoperated cataracts were nuclear (n=2404), either pure (n=1489) or mixed (n=915). Cortical cataracts were graded in 512 participants (195 pure and 317 mixed), and PSCs were graded in 1084 participants (235 pure and 849 mixed). The use of any medication or supplement was reported by 13.8% (n=668). Few participants (n=69, 1.2%) reported taking any nutritional supplements; 21 of these self-reported the use of a supplement, and 48 brought the supplement to the clinical examination. One third of supplement users were taking a multivitamin supplement (n=25), and 42% (n=29) took a vitamin B complex. Dietary measures of vitamin C were available in 5535 of those with plasma vitamin C.

## Plasma Vitamin C

In univariable analysis, there were significant inverse trends with vitamin C and increasing age, male gender, lowest socioeconomic group, highest quartile of outdoor exposure, current tobacco use, current use of biomass cooking fuel, and indices of poor nutrition (BMI <18.5 or MUAC <22 in men and <20 in women). Alcohol use was rarely reported by women (n=12, 1.1%). There was no association between current alcohol consumption and vitamin C in men. Being overweight (BMI >25) or having diabetes, higher levels of cholesterol, and increasing diastolic blood pressure were associated with increasing vitamin C. Dietary vitamin C intakes increased with increasing plasma vitamin C.

In age- and sex-adjusted analyses, there were strong inverse associations with increasing plasma vitamin C and any cataract (operated or unoperated) in both centers (Table 1). These associations were only slightly attenuated in further analyses that also included tobacco use, current fuels, BMI, MUAC, diastolic blood pressure, outdoor exposure, diabetes, and socioeconomic status. The pattern of an inverse association with vitamin C was similar for nuclear, cortical, and PSC cataracts (Table 2, available at http://aaojournal.org). There were no significant (*P* < 0.05) interactions of vitamin C and study center in the association with cataract or with type of cataract. However, for nuclear and PSC cataract, there was some suggestion (*P* < 0.1) that the association with vitamin C was steeper in the north compared with the south.

## Associations with Cataract, Plasma Vitamin C, and Other Blood Antioxidants

In univariable analyses adjusting only for age, sex, and study center, there were significant inverse associations between quartiles of retinol, α-tocopherol, β-carotene, lutein, or zeaxanthin and any cataract (data not shown). In multivariable analyses including all antioxidants in the same model (lutein or zeaxanthin entered separately), the associations of any cataract with α-tocopherol and β-carotene were markedly reduced and not significant (Table 3). Inverse associations remained for all other antioxidants, although the strength of the associations was reduced. In contrast, for vitamin C the inclusion of the other antioxidants made only a small attenuation to the association with any cataract. In multivariable analyses by type of cataract, strong inverse associations remained for vitamin C with nuclear, cortical, and PSC, with the steepest slope observed for PSC (Table 4, available at http://aaojournal.org). No other antioxidants were associated with cortical cataract. Lutein, zeaxanthin, and β-carotene were not associated with PSC, whereas there was a weak association with α-tocopherol and PSC (*P* < 0.04) and retinol and PSC (*P* < 0.01). There were weak associations with lutein and retinol with nuclear cataracts. There were no significant interactions by center in any models.

## Association with Cataract and Dietary Vitamin C

In contrast with plasma vitamin C, dietary vitamin C intakes were similar in women compared with men. In univariable analysis of factors associated with dietary vitamin C, there were inverse significant associations with age, SES, current tobacco use, low BMI, and current use of biomass cooking fuel, and positive associations with diabetes, being overweight (BMI ≥25), diastolic blood pressure, and cholesterol. There was no association with MUAC (*P*=0.4).

In univariable and multivariable analyses, inverse associations were found between dietary vitamin C and any cataract (Table 5). Further analyses by type of cataract showed similar results for all types of cataract (data not shown). There was no interaction between dietary vitamin C and study center in any models.

## Discussion

We found a strong inverse association between vitamin C and cataract. The results confirm those from our previous feasibility study.^6^ The present study was 5 times larger, included participants from both north and south India, and added dietary measures of vitamin C. To our knowledge, this is the first large population-based study to provide evidence from a low- or middle-income country on vitamin C and cataract.

Plasma vitamin C levels in our study were low. We think it unlikely that the low values were due to degradation of the samples. Considerable care was taken in the collection, processing, and transport of the samples, and our assay was carefully standardized. Moreover, the results show typical patterns observed in other studies, such as lower levels in men and tobacco users.^14^ It is unlikely that these patterns would be preserved if the blood samples had degraded. The few studies that have measured plasma vitamin C levels in India, including our feasibility study, also reported low levels.^6,15,16^ In a population aged 20–50 years, the mean plasma vitamin C was 18 μmol/L and 74% had values <22.7 μmol/L. In a case-control study of cataract in an older age group (50–70 years), the mean plasma vitamin C levels were ∼13 μmol/L in controls.

Epidemiologic studies have examined associations with cataract and vitamin C, but there is limited evidence from India.^16,17^ The US-India case-control study conducted in the northern part of India found an unexpected positive association between plasma vitamin C and the combination of nuclear with PSC cataract; however, an antioxidant index estimated from glutathione peroxidase, ascorbic acid, vitamin E, and glucose-6-phosphate dehydrogenase was strongly inversely associated.^17^ In a case-control study from western India, plasma ascorbic acid was lower in low-income cataract cases compared with low-income controls; it was unclear which, if any, potential confounders were included in the analysis.^16^ In other epidemiologic studies, predominantly conducted in high-income countries, plasma vitamin C levels were substantially higher than in our study. For example, in 3 studies that reported a significant inverse association with plasma vitamin C and cataract (2 from southern Europe and 1 from the United States), the lowest quintile of plasma vitamin C was <50 μmol/L^18,19^ or <30 μmol/L,^20^ and the highest quintile was approximately 70 μmol/L^19,20^ or >86 μmol/L in the Nutrition and Vision project^18^ (possibly reflecting that the study was conducted only in women). By comparison in the India Age-Related Eye Disease Study, the lowest and highest quintile cut points were <2 and >21 μmol/L. In the National Health and Nutrition Examination Survey, a 1 mg/dL increase in serum vitamin C (equivalent to a 58 μmol/L increase) was associated with a 26% reduction in self-reported cataract.^21^ The average vitamin C levels were 62 μmol/L (standard deviation = 28). In our study, the association of vitamin C with cataract was across the range of vitamin C levels in our population and not found only for those with clinical deficiency (<11 μmol/L). The finding of gradients of risk across different levels of plasma vitamin C suggests that, at least within the range of vitamin C levels in the studies, there is no clear threshold above which there is no additional benefit or indeed adverse effect. These comments are tentative because of the relative paucity of studies that have investigated plasma vitamin C and cataract and the inconsistent findings in results from high-income countries. No association between plasma vitamin C and cataract was found in the Pathologies Oculaires Liées à l'Age (POLA) study in southern France (mean vitamin C ∼35 μmol/L)^22^ or in an older age group in the United Kingdom (OR for nuclear cataract = 1.0 [95% CI, 0.5–1.9] for levels <24 μmol/L compared with >57 μmol/L).^23^ In the Baltimore Longitudinal Study on Aging in the United States, no association was found with nuclear (OR 1.31; 95% CI, 0.65–2.60) or cortical cataract with plasma vitamin C (<60 μmol/L) compared with high levels (>82 μmol/L).^24^ In a study in a Hong Kong fishing community, no significant association was found between plasma vitamin C <40 μmol/L compared with >80 μmol/L and either early (OR 0.7; 95% CI, 0.3–1.6) or late cataract (OR 0.8; 95% CI, 0.2–3.2).^25^ Many of these studies were small, for example with less than 500 participants,^18,23–25^ and low powered to detect other than large effects.

The associations with plasma vitamin C and cataract were attenuated slightly when other blood antioxidants were included in the model. Inverse associations with cataract remained for lutein, zeaxanthin, and retinol, but not for β-carotene or α-tocopherol. Evidence from other studies on these blood antioxidants is relatively sparse, especially from studies that included also vitamin C. Of the studies that investigated β-carotene,^19,20,23,24^ only one^23^ found an inverse association, but this study did not adjust for other antioxidants. Two studies investigated plasma lutein and zeaxanthin.^23,26^ Only one, the POLA study, found a significant inverse association with zeaxanthin and no association for lutein, although the OR for nuclear cataract was in the direction of benefit (OR 0.6).^26^ The POLA study was also the only study to report a significant inverse association with retinol, whereas 3 other studies found no association.^19,20,24^ Lutein and zeaxanthin levels in the POLA study were comparable to those in our study, but retinol levels were higher. Of the studies that have investigated α-tocopherol,^19,20,23,24,27^ only one reported an inverse association with nuclear cataract,^24^ whereas higher ORs between 2 and 3 were associated with high levels of α-tocopherol and cortical and PSC opacities in the Italian American Study.^20^ In all these studies, the levels of α-tocopherol were higher than in our participants. Other studies have also investigated associations with blood carotenoids or tocopherols, with some studies reporting inverse associations for lutein and zeaxanthin^28^ or α-tocopherol^29–31^ or all tocopherols.^32^ However, none of these studies measured blood vitamin C and could not evaluate the independent association with these other antioxidants.

We examined associations by type of cataract. Associations with vitamin C remained strong for all types of cataract irrespective of whether other antioxidants were included in the models. The associations were stronger for vitamin C and PSC cataract, but the large number of people with mixed cataracts, especially nuclear cataracts, limits the interpretation of these results. In analyses of vitamin C alone, there was some evidence that the association was stronger in north India compared with south India (*P* for interaction ∼0.1 for nuclear and PSC cataract). The interaction was weaker when other antioxidants were included.

There is strong biological plausibility for the importance of vitamin C in the lens. Vitamin C is found at concentrations in lens or aqueous of approximately 20- to 30-fold that of the plasma^33^ and even higher in the vitreous.^34^ Early studies in India were among the first to report that vitamin C concentrations measured in the aqueous of patients undergoing cataract extraction were higher in those with normal lenses compared with those with mature cataracts.^35^ The authors also noted that aqueous vitamin C levels in the Indian studies were considerably lower than those reported for equivalent studies on vitamin C in aqueous in western populations. Vitamin C is a powerful reducing agent and protects the lens from oxidative stress. Lutein, zeaxanthin, and α-tocopherol have been detected in human lenses but at similar levels to plasma, whereas α-carotene, β-carotene, lycopene, and β-cryptoxanthin have not been detected.^36^ Vitamin C acts synergistically with vitamin E, and both vitamins C and E maintain the antioxidant activity of glutathione.^37^

Randomized controlled trials (RCTs) of antioxidant supplementation have shown largely negative results on cataract.^38^ Single vitamin supplementation trials found no benefit from vitamin E alone^39,40^ or from β-carotene alone.^41,42^ Two placebo-controlled factorial trials found no effect of either vitamin E and vitamin C^43^ or α-tocopherol and β-carotene.^44^ There was no benefit from high-dose multivitamin supplement (vitamins C and E, and β-carotene) in the Age-Related Eye Disease Study.^45^ An RCT in Italy with a longer follow-up than most trials found a reduced rate of progression of nuclear opacities after an average 9 years of a broad multivitamin/mineral supplement use;^46^ that trial also found an increase in PSC opacities. One RCT has been undertaken in India and reported no benefit in the rate of progression of opacities over a 5-year period from supplementation with high-dose vitamins A, C, and E.^47^ The trial was small (n=798), and although it was powered to detect a small change in nuclear opacity, the use of clinical grading at the slit lamp may have led to random errors in grading. The study duration also may have been too short.

In our study, dietary intakes of vitamin C also showed an inverse association with cataract. In high-income countries, associations with dietary vitamin C and cataract have been reported in some studies^18,19,48,49^ but not in others.^50,51^ There are no comparable data on associations with cataract and dietary vitamin C in India or other low- or middle-income countries. Caution is required in comparing dietary intake levels across studies because of the variations in dietary assessment methods and food composition tables. We used the ICMR food composition tables, which provide values of vitamin C from food items common to the Indian population.

In our study, measurement of diet and blood antioxidants were made at the same time as determination of lens status, making it more difficult to establish the temporal relationship between vitamin C and cataract. There is no evidence that cataract can lead to a change in blood antioxidant level for biological reasons, nor is it likely that plasma vitamin C status was influenced by supplementation because use of any nutritional supplement was rare in our participants. We cannot exclude the possibility that reduced household income due to a family member with cataract may have influenced diet. Other limitations in our study include a single measurement of vitamin C and dietary intake increasing the possibility of measurement error diluting the associations. We attempted to minimize recall error by using a method that made little demand on participants because they were only asked to describe the meals in the previous 24 hours. Other dietary methods such as 3- or 5-day weighed intakes are preferable measures of usual intakes but are more resource intensive, whereas food-frequency questionnaires are relatively novel in the Indian setting. We had no information from the ICMR tables of dietary intakes of lutein and zeaxanthin or retinol and could not assess the association of dietary intakes of these nutrients. The strengths of our study include the large sample size and precision of our results, the high response rates, the recruitment of participants from 2 different geographic areas of India, the collection of information on a wide range of potential confounders, the accurate measurement of cataract through lens photography, and the quality assurance in grading.

In conclusion, the strong association with vitamin C and cataract in our vitamin C–depleted population may, in part, explain the high levels of cataract in India. Studies are needed on vitamin C and cataract in other vitamin C–depleted populations.

## Figures and Tables

**Table 1:** Association of Cataract with Plasma Vitamin C

Any Cataract or Operated Cataract	North India		South India		Both Centers	
	Model 1⁎	Model 2†	Model 1⁎	Model 2†	Model 1⁎	Model 2†
Vitamin C (μmol/L)						
Q1: All < 2	1	1	1	1	1	1
Q2: 4.4 (3.1–5.7)†						
OR	0.70	0.74	0.84	0.84	0.74	0.76
95% CI	0.53–0.94	0.56–0.99	0.63–1.13	0.63–1.12	0.60–0.91	0.62–0.94
Q3: 12.7 (9.9–16.2)‡						
OR	0.56	0.62	0.81	0.86	0.66	0.72
95% CI	0.42–0.75	0.45–0.84	0.64–1.04	0.67–1.10	0.54–0.80	0.59–0.87
Q4: 38.4 (28.2–52.5)‡						
OR	0.48	0.55	0.63	0.71	0.54	0.61
95% CI	0.36–0.65	0.41–0.74	0.50–0.80	0.57–0.89	0.45–0.65	0.51–0.74
*P* trend§	3.2×10^−7^	0.00003	0.00003	0.004	2.2×10^−10^	1.1×10^−6^
*P* interaction∥					0.3	0.4

CI = confidence interval; OR = odds ratio.

⁎ Adjusted for age and sex.

† Adjusted for age, sex, tobacco use, current fuels, BMI mid-upper arm circumference, diastolic blood pressure, outdoor exposure, diabetes, and socioeconomic status.

‡ Median (interquartile range).

§ Test for trend by design-adjusted Wald test with 4 ordered categories of vitamin C entered as independent continuous variable.

∥ Test for interaction by center and vitamin C by design-adjusted chi-square test.

**Table 2:** Association of Cataract with Plasma Vitamin C

Plasma Vitamin C (μmol/L)	North India		South India		Both Centers	
Nuclear Cataract	Model 1⁎	Model 2†	Model 1⁎	Model 2†	Model 1⁎	Model 2†
Q1	1	1	1	1	1	1
Q2						
OR	0.74	0.79	0.86	0.83	0.76	0.78
95% CI	0.53–1.02	0.58–1.09	0.64–1.17	0.62–1.12	0.60–0.95	0.63–0.98
Q3						
OR	0.51	0.56	0.85	0.89	0.64	0.69
95% CI	0.37–0.70	0.40–0.79	0.66–1.10	0.69–1.14	0.51–0.79	0.56–0.86
Q4						
OR	0.44	0.52	0.62	0.69	0.50	0.58
95% CI	0.32–0.61	0.38–0.72	0.48–0.81	0.54–0.89	0.41–0.62	0.47–0.72
*P* trend§	1.5 × 10^−8^	3.5 × 10^−6^	0.0002	<0.01	8.0 × 10^−11^	4.7 × 10^−7^
*P* interaction∥					0.07	0.12
Cortical cataract						
Q1	1	1	1	1	1	1
Q2						
OR	0.79	0.84	0.87	0.91	0.83	0.88
95% CI	0.52–1.21	0.56–1.28	0.53–1.43	0.56–1.47	0.60–1.14	0.64–1.20
Q3						
OR	0.61	0.64	0.68	0.70	0.65	0.68
95% CI	0.42–0.91	0.43–0.95	0.47–0.99	0.48–1.04	0.50–0.85	0.52–0.89
Q4						
OR	0.66	0.74	0.60	0.63	0.61	0.65
95% CI	0.39–1.11	0.45–1.20	0.44–0.83	0.47–0.86	0.47–0.80	0.50–0.85
*P* trend§	0.02	0.06	0.001	0.003	0.0001	0.001
*P* interaction∥					0.96	0.91
PSC cataract						
Q1	1	1	1	1	1	1
Q2						
OR	0.70	0.70	1.05	1.03	0.79	0.79
95% CI	0.50–0.99	0.49–0.99	0.76–1.45	0.76–1.40	0.61–1.03	0.61–1.02
Q3						
OR	0.55	0.56	0.89	0.93	0.66	0.68
95% CI	0.37–0.82	0.37–0.86	0.67–1.17	0.70–1.22	0.51–0.85	0.52–0.89
Q4						
OR	0.42	0.44	0.68	0.69	0.51	0.53
95% CI	0.30–0.59	0.32–0.61	0.53–0.86	0.54–0.89	0.41–0.64	0.42–0.66
*P* trend§	1.8 × 10^−6^	0.00001	0.001	0.004	1.7 × 10^−8^	2.7 × 10^−7^
*P* interaction∥					0.08	0.10

CI = confidence interval; OR = odds ratio; PSC = posterior subcapsular cataract.

⁎ Adjusted for age and sex.

† Adjusted for age, sex, tobacco use, current fuels, BMI, mid-upper arm circumference, diastolic blood pressure, outdoor exposure, diabetes, and socioeconomic status.

§ Test for trend by design-adjusted Wald test with 4 ordered categories of vitamin C entered as independent continuous variable.

∥ Test for interaction by center and vitamin C by design-adjusted chi-square test.

**Table 3:** Associations of Cataract with Plasma Vitamin C and Other Antioxidants

Blood antioxidants (μmol/L)	Vitamin C	Retinol	β-Carotene	α-Tocopherol	Lutein	Zeaxanthin
Quartiles	OR (95% CI)⁎	OR (95% CI)⁎	OR (95% CI)⁎	OR (95% CI)⁎§	OR (95% CI)⁎	OR (95% CI)⁎
Q1 (reference)	1	1	1	1	1	1
Q2	0.79 (0.65–0.97)	0.68 (0.57–0.81)	1.10 (0.91–1.32)	1.00 (0.85–1.18)	0.98 (0.81–1.18)	0.94 (0.73–1.22)
Q3	0.76 (0.62–0.92)	0.68 (0.56–0.83)	1.13 (0.87–1.47)	0.90 (0.75–1.08)	0.82 (0.67–1.00)	0.91 (0.72–1.16)
Q4	0.68 (0.57–0.82)	0.72 (0.59–0.88)	1.03 (0.83–1.28)	0.91 (0.72–1.14)	0.79 (0.63–0.99)	0.76 (0.58–0.99)
*P* trend†	<0.0001	0.01	0.9	0.3	0.01	0.02
*P* interaction‡	0.5					

CI = confidence interval; OR = odds ratio.

⁎ Adjusted for age, sex, study center, tobacco use, current fuels, body mass index, mid upper arm circumference, diastolic blood pressure, outdoor exposure, diabetes, socioeconomic status, and all other antioxidants in the table. Lutein and zeaxanthin in alternate models. Results for vitamin C, retinol, β-carotene, and α-tocopherol shown for models with lutein. Results (not shown) with zeaxanthin replacing lutein in model almost identical to result with lutein.

† Test for trend by design-adjusted Wald test with 4 ordered categories of antioxidant entered as independent continuous variable.

‡ Test for interaction of vitamin C and study center by design-adjusted chi-square test.

§ Levels of α-tocopherol were adjusted for cholesterol.

**Table 4:** Associations of Cataract Type with Plasma Vitamin C and Other Antioxidants∥

Blood Antioxidants	Any Nuclear Cataract	Any Cortical Cataract	Any PSC
	OR (95% CI)⁎	OR (95% CI)⁎	OR (95% CI)⁎
Plasma vitamin C			
Q1: All < 2	1	1	1
Q2: 4.4 (3.1–5.7)†	0.81 (0.65–1.01)	0.88 (0.64–1.21)	0.79 (0.62–1.02)
Q3: 12.7(9.9–16.2)†	0.74 (0.60–0.92)	0.71 (0.54–0.94)	0.72 (0.54–0.94)
Q4: 38.4 (28.2–52.5)†	0.66 (0.54–0.80)	0.70 (0.54–0.90)	0.58 (0.45–0.74)
* P* trend‡	0.0001	0.002	0.00003
* P* interaction§	0.2	0.9	0.2
Retinol			
Q1: 0.77 (0.61–0.87)†	1	1	1
Q2: 1.12 (1.05–1.21)†	0.65 (0.54–0.78)	0.74 (0.53–1.05)	0.62 (0.47–0.81)
Q3: 1.46 (1.37–1.56)†	0.68 (0.55–0.84)	0.70 (0.49–1.00)	0.62 (0.46–0.83)
Q4: 1.98 (1.80–2.27)†	0.69 (0.56–0.84)	0.78 (0.55–1.10)	0.65 (0.50–0.85)
* P* trend‡	0.04	0.2	0.01
β-carotene			
Q1: 0.06 (0.04–0.08)†	1	1	1
Q2: 0.12 (0.10–0.13)†	1.04 (0.84–1.28)	0.95 (0.68–1.34)	1.19 (0.92–1.53)
Q3: 0.19 (0.17–0.22)†	1.03 (0.79–1.34)	1.07 (0.73–1.55)	1.07 (0.78–1.48)
Q4: 0.36 (0.29–0.47)†	0.89 (0.74–1.07)	0.87 (0.61–1.25)	0.98 (0.70–1.37)
* P* trend‡	0.7	0.6	0.8
α-tocopherol			
Q1: 13.0 (11.1–14.4)†	1	1	1
Q2: 18.0 (16.9–19.1)†	0.89 (0.74–1.07)	0.97 (0.72–1.32)	0.89 (0.74–1.07)
Q3: 22.9 (21.5–24.7)†	0.82 (0.66–1.00)	0.74 (0.52–1.05)	0.78 (0.60–1.03)
Q4: 32.3 (29.0–37.4)†	0.80 (0.62–1.05)	0.86 (0.57–1.32)	0.74 (0.99–1.72)
* P* trend‡^,^∥	0.09	0.4	0.04
Lutein			
Q1: 0.125 (0.098–0.147)†	1	1	1
Q2: 0.197 (0.181–0.217)†	0.92 (0.75–1.12)	1.29 (0.92–1.83)	1.05 (0.81–1.37)
Q3: 0.281 (0.255–0.308)†	0.82 (0.66–1.03)	0.99 (0.70–1.42)	0.91 (0.68–1.20)
Q4: 0.460 (0.393–0.586)†	0.77 (0.60–1.01)	1.06 (0.70–1.61)	0.92 (0.68–1.26)
* P* trend†	0.04	0.8	0.5
Zeaxanthin			
Q1: 0.021 (0.016–0.025)†	1	1	1
Q2: 0.033 (0.030–0.036)†	0.93 (0.71–1.22)	1.17 (0.76–1.78)	0.99 (0.74–1.32)
Q3: 0.045 (0.041–0.048)†	0.97 (0.74–1.26)	0.97 (0.64–1.48)	1.03 (0.77–1.38)
Q4: 0.066 (0.058–0.080)†	0.80 (0.60–1.06)	0.86 (0.51–1.44)	0.86 (0.63–1.16)
* P* trend‡	0.1	0.3	0.3

CI = confidence interval; OR = odds ratio; PSC = posterior subcapsular cataract.

⁎ Adjusted for age, sex, study center, tobacco use, current fuels, body mass index, mid upper arm circumference, diastolic blood pressure, outdoor exposure, diabetes, socioeconomic status, and all other antioxidants in the table. Lutein and zeaxanthin in alternate models. Results for vitamin C, retinol, β-carotene, and α-tocopherol shown for models with lutein. Results (not shown) with zeaxanthin replacing lutein in model almost identical to result with lutein.

† Median (interquartile range).

‡ Test for trend by design-adjusted Wald test with 4 ordered categories of antioxidant entered as independent continuous variable.

§ Test for interaction of vitamin C and study center by design-adjusted chi-square test.

∥ Levels of α-tocopherol were adjusted for cholesterol in the analysis.

**Table 5:** Association with Dietary Vitamin C and Cataract

Dietary Vitamin C (mg/day)	OR (95% CI)⁎	OR (95% CI)†
Q1: 10.8 (8.0–13.1)‡	1	1
Q2: 19.6 (17.6–21.7)‡	0.83 (0.64–1.08)	0.85 (0.65–1.11)
Q3: 28.6 (26.0–31.2)‡	0.87 (0.67–1.13)	0.90 (0.69–1.17)
Q4: 40.2 (36.8–44.0)‡	0.71 (0.53–0.94)	0.74 (0.57–0.96)
Q5: 66.4 (55.8–83.9) ‡	0.72 (0.58–0.90)	0.78 (0.62–0.98)
*P* trend§	0.001	0.006
*P* interaction∥	0.7	0.8

CI = confidence interval; OR = odds ratio.

⁎ Adjusted for age, sex, dietary energy intake, study center.

† Adjusted for age, sex, dietary energy intake, study center, tobacco use, current fuels, BMI, diastolic blood pressure, outdoor exposure, diabetes, and socioeconomic status.

‡ Median (interquartile range).

§ Test for trend by design-adjusted Wald test with 4 ordered categories of antioxidant entered as independent continuous variable.

∥ Test for interaction of dietary vitamin C and study center by design-adjusted chi-square test.
